# Startle Increases the Incidence of Anticipatory Muscle Activations but Does Not Change the Task-Specific Muscle Onset for Patients After Subacute Stroke

**DOI:** 10.3389/fneur.2021.789176

**Published:** 2022-01-13

**Authors:** Nan Xia, Chang He, Yang-An Li, Minghui Gu, Zejian Chen, Xiupan Wei, Jiang Xu, Xiaolin Huang

**Affiliations:** ^1^Department of Rehabilitation Medicine, Tongji Hospital, Tongji Medical College, Huazhong University of Science and Technology, Wuhan, China; ^2^World Health Organization Collaborating Centre for Training and Research in Rehabilitation, Wuhan, China; ^3^State Key Lab of Digital Manufacturing Equipment and Technology, Institute of Rehabilitation and Medical Robotics, Huazhong University of Science and Technology, Wuhan, China

**Keywords:** startle, stroke, anticipatory muscle activation, rehabilitation, anticipatory postural adjustments

## Abstract

**Objectives:** To demonstrate the task-specificities of anticipatory muscle activations (AMAs) among different forward-reaching tasks and to explore the StartleReact Effect (SE) on AMAs in occurrence proportions, AMA onset latency or amplitude within these tasks in both healthy and stroke population.

**Methods:** Ten healthy and ten stroke subjects were recruited. Participants were asked to complete the three forward-reaching tasks (reaching, reaching to grasp a ball or cup) on the left and right hand, respectively, with two different starting signals (warning-Go, 80 dB and warning-startle, 114 dB). The surface electromyography of anterior deltoid (AD), flexor carpi radialis (FCR), and extensor carpi radialis (ECR) on the moving side was recorded together with signals from bilateral sternocleidomastoid muscles (SCM), lower trapezius (LT), latissimus dorsi (LD), and tibialis anterior (TA). Proportions of valid trials, the incidence of SE, AMA incidence of each muscle, and their onset latency and amplitude were involved in analyses. The differences of these variables across different move sides (healthy, non-paretic, and paretic), normal or startle conditions, and the three tasks were explored. The ECR AMA onset was selected to further explore the SE on the incidence of AMAs.

**Results:** Comparisons between move sides revealed a widespread AMA dysfunction in subacute stroke survivors, which was manifested as lower AMA onset incidence, changed onset latency, and smaller amplitude of AMAs in bilateral muscles. However, a significant effect of different tasks was only observed in AMA onset latency of muscle ECR (*F* = 3.56, *p* = 0.03, *η*^2^_*p*_ = 0.011), but the significance disappeared in the subsequent analysis of the stroke subjects only (*p* > 0.05). Moreover, the following *post-hoc* comparison indicated significant early AMA onsets of ECR in task cup when comparing with reach (*p* < 0.01). For different stimuli conditions, a significance was only revealed on shortened premotor reaction time under startle for all participants (*F* = 60.68, *p* < 0.001, $\eta_{p}^{2}$ = 0.056). Furthermore, stroke survivors had a significantly lower incidence of SE than healthy subjects under startle (*p* < 0.01). But all performed a higher incidence of ECR AMA onset (*p* < 0.05) than with normal signal. In addition, the incidence of ECR AMAs of both non-paretic and paretic sides could be increased significantly *via* startle (*p* ≤ 0.02).

**Conclusions:** Healthy people have task-specific AMAs of muscle ECR when they perform forward-reaching tasks with different hand manipulations. However, this task-specific adjustment is lost in subacute stroke survivors. SE can improve the incidence of AMAs for all subjects in the forward-reaching tasks involving precision manipulations, but not change AMA onset latency and amplitude.

## Introduction

Anticipatory muscle activations (AMAs) are considered as unconscious muscular activities to prevent the upcoming external disturbance to posture brought by the focal movement (1). For example, when we sit at the table and prepare to enjoy a glass of wine, we raise our right shoulder and arm to accurately grasp the glass in front of us. Before the arms are raised, our body will first move back to offset the disturbance of the forward movement, and approximately 50 ms before or at the same time, the muscles of the trunk and limbs have also been activated to prevent disturbance caused by voluntary movement and ensure the precision of grasp action. Such posture adjustments and muscle activation of the trunk and proximal joints are called anticipatory postural adjustments (APAs) and AMAs, respectively. As one motor control strategy, such anticipations are integrated into motor programing in a feed-forward way to both maintain postural stability and improve the focal motor performance (2, 3). The APAs could be observed and evaluated by the displacement of the body center of mass *via* postural graphic analysis and AMAs *via* surface electromyography (sEMG) (3–5) in the corresponding time window (6). In coping with disturbances and task accuracy (7), the AMAs show orderly activations and adjustments of systemic bilateral muscles and were further depicted as a servo-system responding to external or internal disturbance (2).

Compared with the displacement of the body center of mass, which represents the feedforward trunk movements, the AMAs of the whole body can provide more abundant muscle onset information, and it has been used for the movement prediction (3, 8). Related researches on the upper-limb movement at a sitting position indicated that AMA order, the amplitude of contraction, and duration could be used as predictive factors for the forthcoming action (3). The changes in AMA patterns brought about by the limb movements in different directions and speeds are easy to understand due to the different amplitude and directions of the disturbances. Indeed, even at the forearm level, the AMA tune of limb muscles could be opposite when performing finger taps with hand prone or supine (9). This phenomenon suggests that the AMAs are involved in the precision movement of the hand (9), and the timing and sequence of muscle activations can be specifically adjusted according to the position of the forearm. And this intra-limb AMA pattern seems to be further optimized in the precision movement on the dominant hand (7). The above results suggest that with the mastery and proficiency of precision tasks, task-specific AMAs will also be formed simultaneously. Therefore, it is reasonable to believe that skilled activities of hand have developed their specific AMA patterns. In addition, the highly developed human motor cortex and the extensive representative areas of the hand further provide a structural basis for the specialization of the precision movements of the hand. An experiment on monkeys indicated that firing neurons in the motor cortex were significantly different during the execution of reach and grasp (10). Thus, the human motor cortex is very likely to show these task-specific neuron activations, which represent different motor commands, respectively. Increasing pieces of evidence revealed that AMAs and the recruitment of prime mover shared the same motor command (11, 12). This means that the AMA patterns of the precision reach and grasp activities in humans may be task-specific. However, most previous studies using fast-arm flexion, reaching or pointing as their test paradigm (3), rarely consider hand manipulations. Whether such task-specific AMA patterns exist in the forward-reaching tasks involving finger movements is still unknown. This exploration of task-specific AMAs may help the development of motor intention recognition for the arm-hand tasks and provide personalized neurofeedback rehabilitation training for patients with motor dysfunction of the upper limb.

For most stroke survivors, the recovery of arm-hand function is one of the longest and most challenging topics. On the one hand, damage to the central nervous system directly leads to the loss of control of hand movements. On the other hand, abnormal muscle synergy patterns (typically flexor synergy) (13) and spasticity (14) that appear in the upper limbs during the recovery process can also hinder normal arm-hand movements. In addition, even if the single joint movements of the wrist and fingers can be restored, a lot of effort is still needed to regain the dexterity of functional arm-hand movements, such as precision grasp (15). A large number of studies have confirmed that APA training helps to restore the trunk function and balance of patients with stroke (16, 17). Given the important role of AMAs in upper-limb activities, we have reason to believe that targeted training based on AMAs may help the recovery of forearm and hand function in patients with stroke. However, before that, we need to learn more about the AMA patterns in the forearm-hand movement of patients with stroke.

In the stroke population, delayed or even abnormal AMAs occur commonly in global muscles (18) and manifest a systemic less response to the upcoming disturbances, with more severe impairment to the contralateral side (19). These abnormalities require more voluntary compensation from both trunk and limb muscles to counteract disturbances and even ensure the accuracy of movement (6). Unfortunately, these pathological movement patterns involving atypical feedforward AMAs along with abnormal neuromodulation pathways would be over-enhanced with the time past for the severely injured stroke population (20–22). Therefore, evaluating the AMAs early after the stroke onset may allow us to more clearly find the functional defects before the occurrence of overcompensation. And the targeted training of AMAs for the arm-hand movement may be more effective.

Nevertheless, unresponsive AMAs in the stroke population may be submerged in the abnormal background noise and hard to detect (23). Besides the improvement from an sEMG signal processing technology (24), the introduction of the StartReact Effect (SE) (25) may provide a novel approach to trigger AMAs. A loud acoustic stimulus approximately higher than 110 dB could lead to the early initiation of prepared movements, including AMAs (4, 26). This phenomenon is explained as a well-prepared program that can be triggered output in advance mainly through the cortical reticulospinal tract (25). Coincidentally, the functional neuroregulatory structures of APAs include supplementary motor area (27), primary motor cortex (28), and pontomedullary reticular formation (29), which have a high degree of overlap with cortical reticulospinal formation. In healthy subjects, the loud acoustic stimulus has been confirmed to increase the response frequency of AMAs during gait initiation (4). Moreover, some studies further revealed that the exposure to startling does not impact the upper-limb voluntary activities but enhances the actual reaching performance in stroke subjects (30, 31). However, not all movement initiation can be triggered by SE early, it is negative in some distal finger manipulation activities (32). Whether startle has similar effects on reaching tasks involving precision hand movements remains unclear.

In the present study, we have designed a series of goal-directed hand manipulation tasks based on the forward-reaching paradigm (33–35) at a sitting position with the torso fully involved. We hypothesize that there is likely to be specific AMAs in different hand manipulation tasks, and patients with stroke may retain or lose these specificities. Meanwhile, positive SE may exist in these tasks and has a significant impact on the AMAs of patients with stroke. The first aim of our study was to investigate the task specificities of AMAs among different hand activities and further probe into its preservation or atypical changes in the stroke population. Simultaneously, we explored the SE on the occurrence proportion, muscle onset latency, and amplitude of AMAs on trunk and limb muscles in both healthy and stroke participants. The results of this study provide a basis for a better understanding of the AMAs in 3-D upper-limb grasp activities and give suggestions to develop novel approaches to APA rehabilitation after stroke based on SE.

## Materials and Methods

### Participants

Experiments were carried out in age-matched 20 male subjects, 10 healthy volunteers, and 10 patients with stroke. The inclusion criteria for stroke subjects were: (1) first onset of ischemic or hemorrhage of the unilateral cortex and sub-cortex at the subacute phase (7 days to 6 months) (36); (2) age between 18 and 65 years old; (3) able to perform at least 30-degree shoulder flexion with the paretic arm without support at the sitting position; (4) no hearing impairment and able to understand movement instructions; and (5) good tolerance for 114 dB sudden audio stimulation. Participants would be excluded if they suffer impairments from other diseases or states that severely affect the participant's upper-limb and trunk function, such as fracture, rheumatoid arthritis, amputation, and high fever. Finally, all participants need to sign the informed consent before participation. This study was approved by the institutional ethical committee of Tongji Hospital (No. TJ-IRB20210648) and has completed the online pre-registration for clinical research (No. ChiCTR2100048222).

However, we failed to collect the data from one male patient with stroke, due to the missing anterior deltoid (AD) muscle onset data caused by loosely attached electrodes to the sEMG unit. For stroke subjects #8 and #9, the data of bilateral lower trapezius (LT) and latissimus dorsi (LD) were missing due to the temporary malfunction of electrodes. Finally, data from 10 healthy subjects and 9 patients with stroke were used for the analyses of this study. The baseline characteristics were summarized in Table 1. The mean age of stroke and healthy groups was 42.42 ± 13.26 and 36.50 ± 6.95 years old, respectively. The mean time post-stroke onset for the 9 patients was 2.83 ± 2.11 months.

**Table 1:** Demographic characteristics of the participants.

**Subjects**	**Age (yr.)**	**BMI (kg/m^**2**^)**	**Time from onset (mos.)**	**Dominant hand**	**Paretic side**	**Stroke type**	**Lesion location**	**FMA-UE (/60)**	**MAS (/4)**
#1	48	19.16	5.5	R	R	I	Sub- & Cortical	11	0
#2	60	25.09	0.5	R	L	I	Subcortical	65	0
#3	53	20.52	0.5	R	L	I	Cortical	8	0
#4	41	23.72	5	R	R	H	Sub- & Cortical	13	1^+^
#5	42	23.59	1	R	L	I	Sub- & Cortical	10	0
#6	40	18.01	4	R	L	H	Cortical	32	1^+^
#7	53	26.73	1	R	R	I	Cortical	40	1
#8	18	25.39	3	R	R	H	Sub- & Cortical	61	1
#9	27	18.34	5	R	L	H	Cortical	64	1
#**Stroke group** **(*****n*** **=** **9)**	42.44 (13.26)	22.28 (3.31)	2.83 (2.11)	all R	4R/5L	4H/5I	Cortical: 2 Subcortical: 1 Sub- & Cortical: 4	33.78 (24.63)	1 (Mid.)
**Healthy group** **(*****n*** **=** **10)**	36.50 (6.95)	21.86 (2.55)	N/A	all R	N/A	N/A	N/A	N/A	N/A

*BMI, body mass index; FMA-UE, Fugel-Meyer assessment for upper extremity; MAS, modified Ashworth scale*.

*#Represents the stroke participations. The yr. is same as years old. The mos. represent months. Sub- means sub-cortical lesion of the subject. The I and H represent ischemia and hemorrhagic stroke, respectively. The median score of the 9 stroke subjects was provided as grade 1 (Mid.). N/A means not applicable*.

### Clinical Assessments

In subjects with stroke, the Fugel-Meyer assessment for upper extremity (FMA-UE) (37) was used for the evaluation of the motor function of the upper limb. Moreover, the spasticity was assessed by the modified Ashworth scale on biceps (38) on the paretic side. All patients have impairments in the upper extremity with a mean score of FMA-UE at 33.78. And their median score (Grade 1) of modified Ashworth scale revealed normal or slightly increased spasticity. Age, body mass index, and dominant hands were all comparable in both groups (see Table 1).

### Experimental Procedure

**Figure 1:**
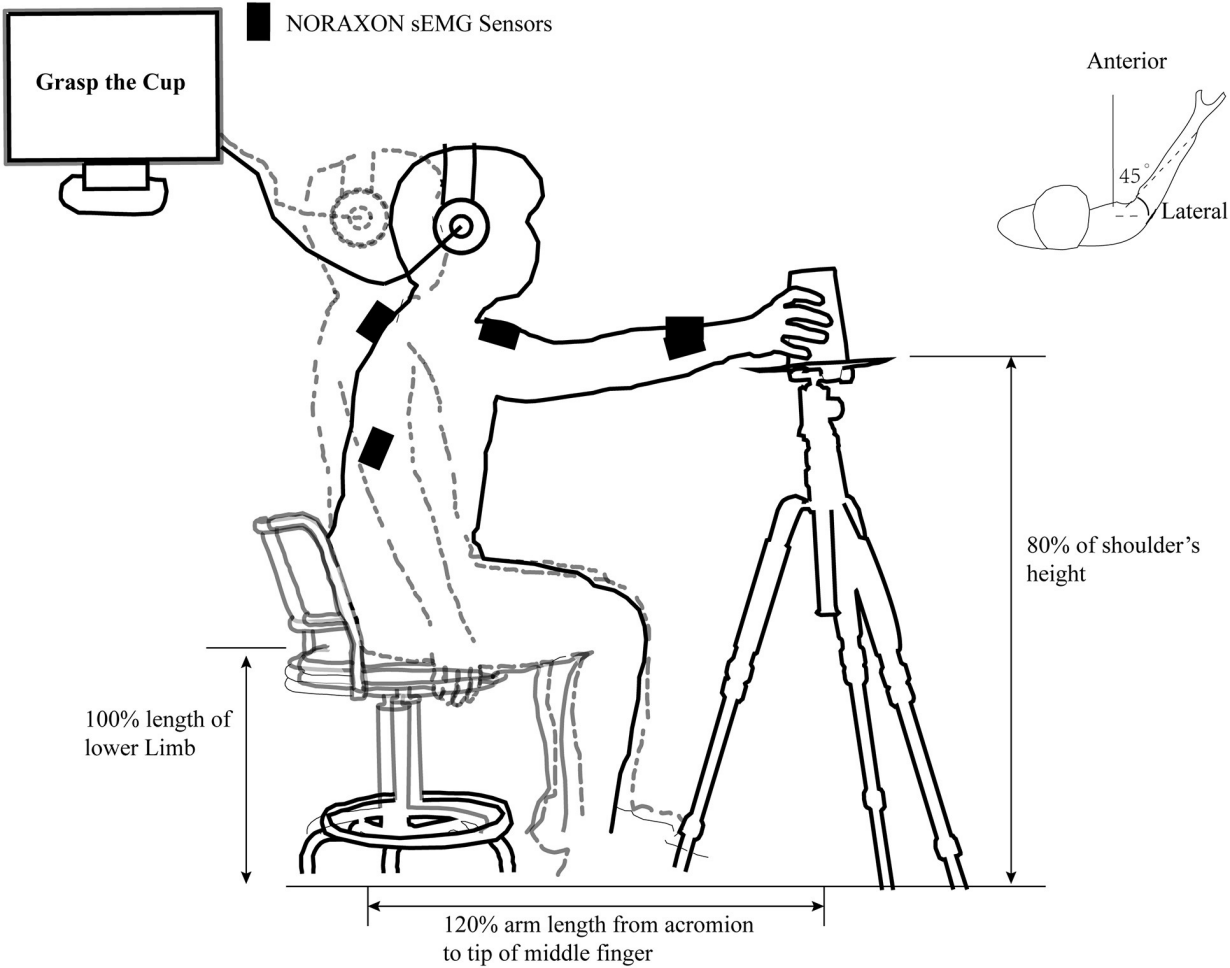
An illustration of the task “reach to grasp a cup” in our study. The outline of the human body in the gray dashed line and the black solid line represent the position of the subject in the pre-motor preparation and motor execution phases in the “reach to grasp a cup” task, respectively. The black rectangle represents the position where the infinite surface EMG sensors is placed. The individually adjusted seat height, the relative position of the cup, and the acromion of movement side are clearly marked. The computer-generated “Warning” and “Go” cues were communicated to the subject through a headset.

In a warm, dry, and quiet room, participants were ordered to sit with their hips and knees flexed ~90° on a height-adjustable seat 1.5 m in front of a blank blackboard. The subject's upper limbs were asked to place next to the trunk as a starting position and keep the whole body relaxed as much as possible. A pallet tripod with 80% shoulder height was placed on the subject's anterolateral side (45°) with a distance of 120% arm length from the acromion to the pallet center. Participants were asked to perform the required task correctly according to the auditory information from a stereophonic headphone (Sennheiser HD25-I; Wedemark, Germany). These tasks include reaching the center of the pallet, reach to grasp a tennis ball (about 58 g), or an inverted coffee takeaway cup with palm inward (7 cm base and 15 cm high) at the same weight. An illustration of the details in the task “reach to grasp a cup” in our study is provided in Figure 1.

The 3 tasks with 10 repetitions of each were randomly assigned into 30 trials within a test. Subjects would take an 1-min break after completing 10 consecutive trials. On each trial, within 5 s after the start of the experimental program, the subjects were verbally informed of the target action, such as “reaching to grasp the cup,” and then hear a warning cue with continuous “beep” (82 dB, 1,000 Hz) for 0.5 s to prompt for “Get ready.” In the next 2.5 to 3 s, the second auditory cue “Go” represents the initiation of the aiming task. Intervals between the two cues were separated randomly to prevent anticipation. In control trials, the “Go” cue was a 40-ms “beep” sound as same as the warning cue, whereas it was replaced with a broadband white noise (114 dB, 40 ms) for “Go” in the startle trials. Although some research suggests that 124-dB acoustic stimulus may be more efficient (39) because of patient acceptability, we chose a relatively mild startle stimulation used in similar researches (40, 41). There is a 15-s interval between every two trials to ensure that the subjects are fully relaxed and provide sufficient time for the replacement of the ball or cup on the tripod. About 30 trials were required for the test on each side for all participants, and half of those trials would use startle white noise as a “Go” cue. The orders of 15 control or 15 startling “Go” cues were also randomized in the test. At the beginning of these trials, 5–10 practices were implemented to ensure the smooth completion of the task. Healthy subjects were tested on the right hand first, and stroke participants completed it on the non-paretic side before using the paretic arm. Regardless of the performance, the test operators encouraged all subjects to complete the corresponding task as quickly and accurately as possible throughout the test. Additional encouragement for stroke subjects with obvious muscle paralysis would also be provided. The Psychtoolbox-3 toolkit based on MATLAB (2017b, MathWorks, Natick, MA, USA) was used for the design and execution of all tests.

### Surface Electromyography and Data Preprocessing

The Ag/AgCl surface electrodes connecting to wireless sEMG units were placed bilaterally on the subject's right and left sternocleidomastoid muscles (SCM), the bilateral (LT), bilateral (LD), and tibialis anterior (TA). AD, flexor carpi radialis (FCR), and extensor carpi radialis (ECR) of the motion side were also placed with electrodes for sEMG recording. All operations in our experiment followed the SENIAM recommendations, and the signals were visually verified by voluntary contraction. The sEMG signals were collected by the Ultimu EMG system (Noraxon USA Inc., Scottsdale, AZ, USA) at a sampling rate of 2,000 Hz.

The raw data of sEMG were output and processed *via* the MATLAB software (2017b, MathWorks, Natick, MA, USA). The raw data directly output pre-processed first, including data segmentation and data filtering process by using a 30–300 Hz bandpass filter and a 50-Hz notch filter. The Teager–Kaiser energy operation was used to process the filtered data, which was based on the simultaneous sEMG amplitude and instantaneous frequency as a reference basis and has achieved higher reliability than normal methods (24). After data rectification and normalization, the threshold detection method was used to determine the onset time of muscle activation. The threshold *T* can be set as:


$$\begin{array}{r} T=\mu +h\sigma \end{array}$$

where *μ* and *σ* are the mean and SD of baseline amplitudes of the time window 2,500–500 ms before each “Go” cue, and *h* is a preset variable (*h* = 3 in this study). Finally, the morphological operation (42) was used to eliminate false-positive activation points.

According to the video recording, trials with obvious task errors (such as movement initiation before the “Go” cue or performing the wrong task) were eliminated first. The left and right side data of healthy subjects were converted to the same model, and the data of patients with stroke were converted to the non-paretic side and the paretic side data. The muscle activation onset time of AD was considered as time zero (*T*_0_) for all tasks. Moreover, the premotor reaction time was calculated for the interval between the rise of the “Go” signal to *T*_0_. Trials were also excluded from the data analysis if the reaction time of AD is within 30 ms after the “Go” cue or exceeds 400 ms for muscle response. The remaining trials were considered valid. Furthermore, the reaction time of SCM was also calculated. If the reaction time of either SCM was in the time window of 30 to 130 ms after the “Go” cue, the trial will be marked as a positive SCM^+^ trial. SCM^+^ has been suggested as a sign of a complete SE in the upper-limb movements (25). The onset latency of the other muscles each was calculated as the difference between muscle activation onset to *T*_0_. The integrals of sEMG of the muscle activation amplitude were calculated at the APAs window (−100 to +50 ms) (6, 43) to *T*_0_. A detailed description of the definition and calculation process of the parameters and outcome variables were provided in Table 2.

**Table 2:** The definition and calculation process of the parameters and outcome variables.

**Parameters/outcomes**	**Definition**	**Calculation methods**
***T*_0_**	The muscle activation onset time point of anterior deltoid in each trial	Over 10 consecutive samples of the smoothed signal exceeding the threshold (the mean with 3 SD of baseline amplitudes of for anterior deltoid in the time window 2,500 to 500 ms before each “Go” cue).
**Premotor reaction time**	The response time of motor initiation (shoulder flexion)	The interval between the rise of “Go” signal to T_0_ at each trial.
**Valid trials numbers**	Valid trials numbers in the total 60 trials for each subject	Trials were excluded if the premotor reaction time is within 30 ms after the “Go” cue or exceeds 400 ms for muscle response.
**Incidence of SCM^+^**	The percentage of valid startle trials with positive activation of muscle sternocleidomastoid (positive startle react effect) in total valid trails for each subject	“Number of valid startling trials with muscle activation time of either side of bilateral SCMs was in the time window of 30 to 130 ms after ‘Go’ cue”/total valid trails including normal and white noise signals.
**AMA onset**	Anticipatory muscle activation onset of the testing muscles	The onset time point calculation through the threshold method (*T* = μ+*hσ*). In valid trials, the number of muscle activation onset at the APAs window: −100 to + 50 ms to *T*_0_ for each muscle except AD and SCMs.
**Proportion (incidence) of AMAs in valid trials**	The percentage of AMA onset trial numbers in total valid trial numbers for each muscle of each subject	“AMA onset trial numbers for each muscle of each subjects” / “total valid trial numbers for each muscle of each subject”
**Muscle onset latency**	The interval of muscle onset time to *T*_0_ in each trial with positive AMA onset	Use the actual activation time of the target muscle minus the time point of *T*_0_
**AMA amplitude**	The muscle activation amplitude of each muscle at the APA window in each valid trial with AMA onset	The integral of the amplitude of sEMG signal after the preprocessing step in the time window −100 to + 50 ms to *T*_0._

*SCM^+^ represents a positive response of sternocleidomastoid muscle. AMA represents anticipatory muscle activation*.

### Statistical Analyses

Demographic data and proportion variables (e.g., number of valid trials and incidence of SCM^+^) were first checked for their normality of distribution. And independent Student's *t*-tests were used for demographic data comparisons between healthy and stroke subjects. Differences in proportion variables between the two sides of stroke subjects were tested by paired *t*-tests. A general linear model using 3 move sides (healthy vs. non-paretic side vs. paretic side), 2 conditions (startle or normal), and 3 move tasks (reach, reach to grasp a ball or cup) as fixed factors were performed to test the differences. The original model included the main effects of the above 3 fixed factors, interaction effects of each two fixed factors, and a random intercept for subjects. Since no significant effect of interaction terms was found in all tests, they were excluded from the model analysis. Factors without significant main effects were also excluded from the final model for the analyses of corresponding variable. For the final positive models, *post-hoc* comparisons with Bonferroni corrections were used for those fixed factors with significant main effects. Moreover, for the healthy subjects, we made a model involving 2 conditions and 3 move tasks as fixed factors and a random intercept for subjects to do the analysis. Another general linear model using 2 move sides (non-paretic side vs. paretic side), 2 conditions (startle or normal), and 3 move tasks (reach, reach to grasp a ball, or reach to grasp a cup) as fixed factors and including random intercept for subjects was also performed to test the differences in stroke subjects. In the final positive models, *post-hoc* comparisons with Bonferroni corrections were used. The software IBM SPSS 22.0 (IBM Corp., Armonk, NY, USA) was used for all statistical analyses, and *p* < 0.05 was set as the significant level.

## Results

### The Proportion of Valid Trials and Trials With Valid AMA Onset

Those proportion variables were compared between healthy participants and stroke subjects. A total of 1,027 trials were successfully screened out from the tests of 19 participants (1,140 trials). The healthy group had significantly higher numbers of valid trials than the stroke group (55.90 ± 3.28 vs. 52.00 ± 2.60, *F* = 1.02, *p* = 0.01). However, no difference was detected in the numbers of valid trials between the non-paretic and paretic sides (*p* > 0.05) in stroke subjects.

The numbers of valid AMA onset trials of each muscle in the 8 testing muscles range from 418 to 666, and the top two incidences of AMAs were in FCR and ECR as 64.86% and 62.90%, respectively. The contralateral LD (cLT) achieved the lowest at 40.70%. Moreover, the percentage of valid AMA trials in ECR, FCR, and both TAs all indicated lower AMA incidence in the stroke group (*p* < 0.01). No significant differences were detected between the two groups in the proportions of valid AMA trials of bilateral LT and LD (*p* < 0.01). It was worth mentioning that the four patients with lower FMA scores can still detect obvious AMAs in ECR and FCR (see Table 3).

**Table 3:** Proportion of startle responses and anticipatory muscle activations (AMAs) of subjects.

**Subjects**	**Valid trial numbers**	**Incidence of SCM^+^ (%)**	**Proportion of AMAs in valid trials (%)**							
			**ECR**	**FCR**	**iLT**	**cLT**	**iLD**	**cLD**	**iTA**	**cTA**
#1	53	15.09	56.60	52.83	62.26	60.378	58.49	52.83	56.60	32.08
#2	56	23.21	67.86	78.57	82.14	66.07	46.43	41.07	66.07	58.93
#3	52	21.15	61.54	53.85	92.31	65.38	75.00	61.54	50.00	51.92
#4	48	12.50	35.42	35.42	41.67	37.50	33.33	27.08	50.00	37.50
#5	54	7.41	37.04	44.44	59.26	57.41	44.44	55.56	18.52	22.22
#6	54	18.52	51.85	46.30	48.15	40.74	33.33	38.89	48.15	27.78
#7	52	19.23	59.62	71.15	78.85	69.23	38.46	55.77	38.46	40.38
#8	50	20.00	50.00	58.00	N/A	N/A	N/A	N/A	18.00	40.00
#9	49	12.24	34.69	34.69	N/A	N/A	N/A	N/A	24.49	28.57
**#Stroke group**, **mean (SD)**	52.00 (2.60)	16.60 (5.11)	50.51 (12.27)	52.81 (14.88)	66.38 (18.65)	56.67 (12.63)	47.07 (15.11)	47.53 (12.19)	41.14 (17.31)	37.71 (11.82)
**Healthy group**, **mean (SD)**	55.90 (3.28)	25.85 (7.25)	72.69 (9.06)	74.37 (13.51)	69.48 (15.10)	48.18 (7.39)	60.55 (15.66)	48.00 (14.60)	64.36 (20.59)	55.47 (19.50)
***F-*** **value**	1.02	0.39	1.48	0.01	0.78	2.75	0.17	0.02	0.06	2.70
***p-value***	0.01^*^	<0.01^**^	<0.01^**^	<0.01^**^	0.718	0.114	0.105	0.946	<0.01^**^	<0.01^**^

*Incidence of SCM^+^ represents the percentage of numbers of valid trials with position earlier onset of sternocleidomastoid muscle in total valid trial number of subjects. ECR, FCR, i/cLT, i/cLD and i/cTA represents muscle extensor carpi radialis, flexor carpi radialis, ipsilateral/contralateral lower trapezius, ipsilateral/contralateral latissimus dorsi, and ipsilateral/contralateral tibialis anterior*.

* *p-value < 0.05*,

** *p-value < 0.01*.

*# represents the stroke participations*.

### Muscle Onset Latency

In the analyses on the effects of the 3 above fixed factors (move sides, conditions, and tasks) on muscle onset latency, no main effect of conditions was observed (*p* > 0.05). The factor move sides have significant main effects on the activation onset latency of muscle ECR, FCR, bilateral LT, and bilateral TA (*p* < 0.05). No main effects were observed of the 3 fixed factors for the muscle onset latency of bilateral LD (*p* > 0.05). The *post-hoc* comparisons were made on onset latency of the above muscles except LD in the different sides (healthy, non-paretic, and paretic sides). Significant activation delays on muscle ECR, FCR, bilateral LT, and ipsilateral TA (iTA) were observed in the healthy side when compared with the paretic side (*p* < 0.01). Differences of muscle onset latency between the healthy and non-paretic sides were also found in ECR (−5.85 ± 44.23 vs. −17.85 ± 52.80 ms, *p* = 0.04) and contralateral TA (cTA; −20.05 ± 51.76 vs. −41.59 ± 54.11 ms, *p* < 0.01). The muscle onsets of FCR and bilateral LT in the movement of the paretic side were also earlier than the non-paretic side (*p* ≤ 0.02).

The factor move tasks only have significant main effects on the muscle onset latency of ECR (*F* = 3.56, *p* = 0.03, *η*^2^_*p*_ = 0.011) and ipsilateral LT (iLT) (*F* = 3.92, *p* = 0.02, *η*^2^_*p*_ = 0.013). And *post-hoc* comparisons revealed significant differences between the move task reach (ECR, −5.37 ± 45.91; iLT, −8.66 ± 50.03 ms) and cup (ECR, −17.42 ± 50.05; iLT, −22.01 ± 53.69 ms) (*p* < 0.03), indicating an obvious earlier muscle onset of ECR and iLT in the move task cup. However, no differences were detected in onset latency of the above two muscles between task reach and ball (*p* > 0.05).

**Figure 2:**
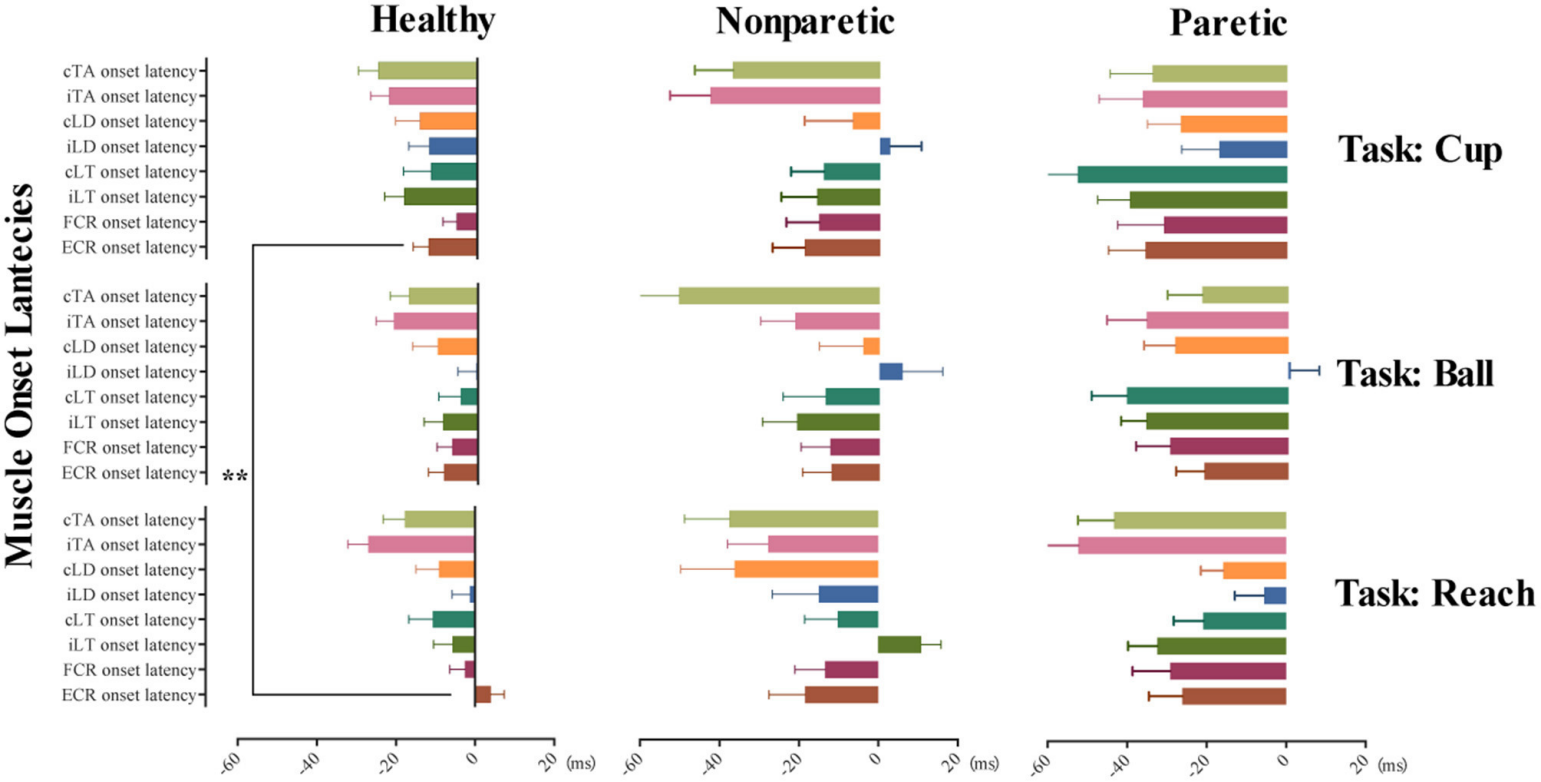
Tunes of AMA onset in the three move tasks of the healthy, non-paretic, and paretic side. This illustrates the tunes of AMA onset in task reach, grasp a ball, and grasp a cup with different move sides, including healthy subjects, non-paretic side, and paretic side of stroke subjects, respectively. Horizontally arranged from left to right are the mean with SEM of AMA onset latency in healthy, non-paretic, and paretic sides; vertically arranged from top to bottom are the different AMA onset latency (mean with SEM) in tasks cup, ball, and reach. **p-value < 0.01.

A further separate general linear model analysis involving fixed factors of conditions and tasks was made for the muscle onset latency of ECR and iLT in healthy subjects. No obvious main effects of conditions were observed for the two variables (*p* > 0.05). And the model was negative for the exploration of the main effect on the conditions, move tasks for muscle onset latency of iLT (*p* > 0.05). Only move tasks have significant main effects on the muscle onset latency of ECR (*F* = 4.98, *p* < 0.01, *η*^2^_*p*_ = 0.024). Moreover, the *post-hoc* comparisons of the above model on different move tasks revealed significant earlier AMA onset of ECR in move task cup when compared to task reach (*p* < 0.01). However, no difference of AMA onset of ECR was detected between task reach and ball or ball and cup (*p* > 0.05). Figure 2 provides mean with SEM of AMA onset latency on each muscle across the 3 move tasks and different move sides.

The general linear model analyses involving factors of move sides, conditions, and tasks for the muscle onset latency of ECR, FCR, bilateral LTs, bilateral LDs, and bilateral TAs of both sides were made in stroke subjects. The results indicated that no main effect of conditions and tasks was observed in the model on the muscle onset latency of all muscles (*p* > 0.05). It also revealed the only significant main effect of move sides for onset latency of FCR (*F* = 5.54, *p* = 0.02, *η*^2^_*p*_ = 0.022), iLT (*F* = 18.86, *p* < 0.01, *η*^2^_*p*_ = 0.072), and cLT (*F* = 12.74, *p* < 0.01, *η*^2^_*p*_ = 0.058). The results revealed a significantly earlier muscle onset of FCR, iLT, and cLT when using the paretic side to complete reaching and grasp move tasks.

### AMA Amplitude of Muscles

In the general linear model analyses, no main effects of conditions on AMA amplitude of these muscles were found (*p* > 0.05). However, significant main effects of factor move sides were observed in the AMA amplitude of ECR (*F* = 8.82, *p* < 0.001, *η*^2^_*p*_ = 0.026), FCR (*F* = 18.09, *p* < 0.001, *η*^2^_*p*_ = 0.052), iLT (*F* = 12.99, *p* < 0.001, *η*^2^_*p*_ = 0.042), cLT (*F* = 7.35, *p* = 0.001, *η*^2^_*p*_ = 0.027), ipsilateral LD (iLD) (*F* = 6.01, *p* = 0.003, *η*^2^_*p*_ = 0.023), and cLD (*F* = 24.33, *p* < 0.001, *η*^2^_*p*_ = 0.099). And the *post-hoc* comparison on move sides revealed the significant smaller AMA amplitude of ECR, FCR, and cLD in both non-paretic and paretic sides of the stroke subjects (*p* < 0.01). The AMA amplitude of cLT on the paretic side was also smaller than healthy side (*p* < 0.01). Nevertheless, significant larger AMA amplitude of iLT and iLD of the paretic side was observed when compared to the non-paretic side (*p* < 0.05). The AMA amplitude of iLT on paretic side also performed larger than healthy side (38.47 ± 35.55 vs. 26.37 ± 27.41 times, *p* < 0.01). The AMA amplitude of iLD on paretic side was equivalent to the healthy side (43.62 ± 35.14 vs. 41.35 ± 36.43 times, *p* > 0.05).

**Figure 3:**
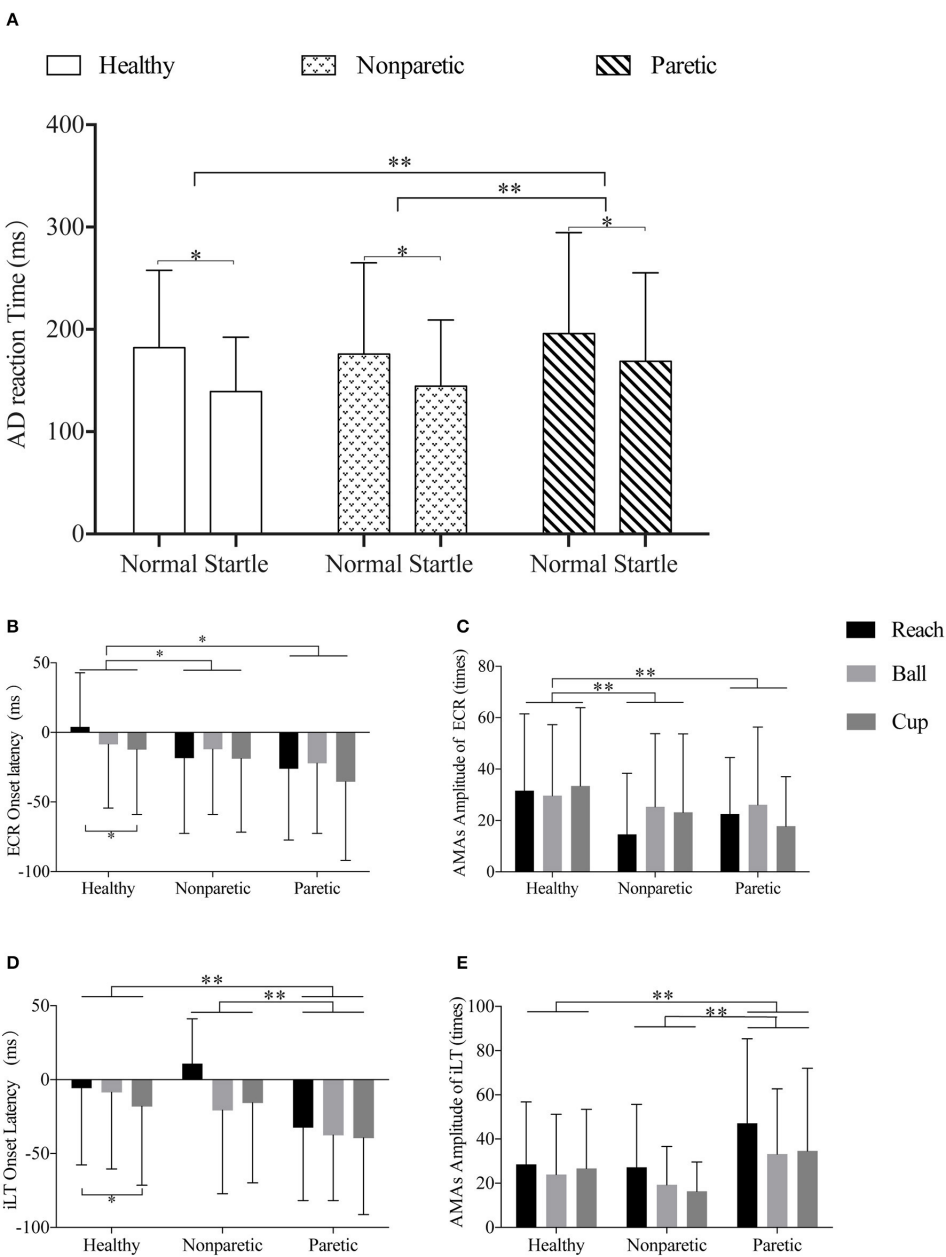
Illustrations of the comparisons across different sides and move tasks in premotor reaction time, muscle onset latency, and amplitude of ECR and iLT. **(A)** Representation of the different AD reaction times of normal or startle conditions of healthy, non-paretic, and paretic sides. **(B,C)** Representation of the different ECR onset latency and ECR AMA amplitude of the “reach,” “ball,” and “cup” tasks in healthy, non-paretic, and paretic sides, respectively. **(D,E)** Representation of the different iLT onset latency and iLT AMA amplitude of the “reach,” “ball,” and “cup” tasks in healthy, non-paretic, and paretic sides, respectively. All data were presented by mean and SD. *p-value < 0.05; **p-value < 0.01.

For the fixed factor of move tasks, only a significant main effect on the AMA amplitude of iLT was observed (*F* = 3.81, *p* = 0.02, *η*^2^_*p*_ = 0.013). And the *post-hoc* analyses on move tasks revealed a larger AMA amplitude of iLT in task reach (32.50 ± 31.66 times) compared with task ball (24.97 ± 26.39 times) (*p* = 0.02). But no difference was detected between reach and cup (26.40 ± 27.96 times) (*p* > 0.05). For AMA amplitude of bilateral TAs, no significant main effects of the three fixed factors (move sides, conditions, and tasks) were detected (*p* > 0.05). The muscle onset latency and AMA amplitude of ECR and iLT are shown as examples in Figures 3B–E. Supplementary Material 1 provides the detailed results of positive general linear models and the *post-hoc* comparisons for these variables.

### Premotor Reaction Time

In the general linear model analyses on all subjects, significant main effects of stimuli conditions (*F* = 60.68, *p* < 0.001, *η*^2^_*p*_ = 0.056) and move sides (*F* = 7.87, *p* < 0.001, *η*^2^_*p*_ = 0.015) were found in the premotor reaction time. However, no main effect of move tasks on premotor reaction time was observed (*p* > 0.05). It was significantly faster in the startle condition (146.47 ± 66.02 ms) than normal condition (183.65 ± 84.56 ms) (*p* < 0.001). And the *post-hoc* comparisons revealed significant differences between the healthy and paretic sides (160.47 ± 68.71 *vs*. 181.51 ± 93.25 ms*, p* = 0.005) and between the non-paretic and paretic sides (159.80 ± 79.03 *vs*. 181.51 ± 93.25 ms*, p* = 0.001). No differences were detected between the healthy and non-paretic sides (*p* > 0.05). The comparisons of AD reaction time of different move sides in startle and normal conditions are shown in Figure 3A.

### Incidence of SCM^+^

In the 525 valid trials with startling “Go” cue, 183 trials were marked as positive SE with SCM^+^ (34.86%). Incidence of SCM^+^ in stroke subjects was significantly lower than healthy participants (*F* = 0.39, *p* < 0.01).

Considering that SE on AMAs is systemic (44), and the percentage of valid AMA trial numbers over 50% would provide better reliability (45), we selected the ECR AMA onset to further explore the SE on the incidence of AMAs. Both the healthy and stroke groups showed a higher incidence of ECR AMA onset in startle than normal condition (*p* < 0.05).

The difference in the incidence of ECR AMA onset in stroke subjects at different conditions of the two sides was further compared. Higher incidence of ECR AMA onset was observed in startle condition on both non-paretic and paretic sides when compared to the normal condition (*t* = −3.21, *p* = 0.01; *t* = −2.83, *p* = 0.02). No difference was detected in the incidence of ECR AMA onset in the startle condition between the two sides of stroke subjects (*p* > 0.05; see Table 3).

## Discussion

In the present study, we first confirmed the existence of task-specific AMA patterns of precision reach and grasp activities at the forearm in the healthy population. At the same time, the AMAs of the proximal joints, trunk, and lower limbs did not show specific adjustments to these hand manipulation tasks. In our test paradigm, a significantly earlier AMA initiation of muscle ECR was observed in tasks with cylinder-shaped grasping movement. However, these task-specific AMAs of ECR disappeared in stroke subjects. Moreover, we found widespread decreased AMA incidence and amplitude on bilateral muscles caused by stroke, which was more pronounced in the limbs on the paretic side. Fortunately, these survivors with severe upper-limb motor impairment still preserved the activation of AMAs in the forearm muscles. Meanwhile, although the patients with stroke performed lower SE incidence, the startle can induce significantly shorter premotor reaction time for all participants in our test paradigm (Figure 3). Startle can also help to increase the incidence of AMAs, but not affect the latency and amplitude of AMAs. In other words, SE leads to the early initiation of AMAs, but it did not affect the corresponding inherent AMA tunes of forward-reaching tasks.

Consistent with previous researches (18, 19), we found systemic AMA dysfunctions in subjects with subacute stroke. And a significant decline of AMA incidence of bilateral muscles in stroke subjects was observed. However, most of the previous studies ignored the fluctuations of AMA response at the individual levels, and the incidence of AMA onset was rarely reported (3, 46, 47). As shown by recent studies, the incidence of AMAs (45, 48, 49) and proportion of SCM^+^ trials (25, 44, 50) were important parameters for the evaluation of APAs or the state of startle-related pathways. It has been suggested in a previous study that stroke-induced atypical APAs *via* an unbalanced excitability/inhibitory control of the central nervous system (51), and typically the system was inhibited (44). Therefore, the proportion variables are important references to the state of this control system. In addition, increasing pieces of evidence suggested the impaired motor programing, and APA deficits were caused by the imbalance or atypical controls of the bilateral cortical reticulospinal tract (44, 51, 52). One pilot study has confirmed the potential benefits in reaching movement performance (31). This improvement in functional performance may have a certain relationship with the response incidence of AMAs. Given the special contribution of the startle to activation of this pathway, some rehabilitation approaches developed based on startle may be helpful for increasing the APA incidence and even improve the arm-hand motor performance.

Compared with the experimental results of Yang et al. (44), the muscles on the non-paretic side did not show large-scale AMA onset abnormalities in our study, which may be due to the choice of different motor test paradigms and participants. Yang uses the reaching task in the standing position as the test paradigm, and does not involve precision hand manipulation. This type of AMAs that do not involve finger movements may be different from precision tasks of the hand. Moreover, our study recruited subjects with subacute stroke, whereas Yang et al. (44) and Pereira et al. (46) recruited participants at the chronic phase from 1 to 20 years after stroke onset. Due to the high adaptability and neuroplasticity of the central nervous system, increasing compensation could be adaptive, which may develop new movements or behavioral patterns based on the remaining neural substrate (36). These changes may strengthen certain abnormal AMA responses (20, 53). It is possible that the non-paretic side of stroke survivors still retains the normal AMA control at the early phase, but this control is replaced by a more compensational one over time. Therefore, a timely understanding of the characteristics of AMA deficit in the subacute phase after stroke may be more helpful to the APA rehabilitation strategies at this stage.

In our research, the ECR AMAs in the cup task showed obvious earlier onset than in the reach task. It is worth mentioning that this task-specific ECR AMA onset was only manifested in normal subjects, and stroke participants did not have this feature. This end-adjustment strategy for different hand activities in ECR AMA onset reflects that our central nervous system tends to adopt remote AMAs to adapt to the execution of various dexterity tasks. This kind of regulation of distal-specific muscles that can improve the task performance has also been found on muscle TA of the anterior leg in fencing athletes (54). Compared with the reaching task, the columnar grasping (task: cup) performs additional wrist flexion and rotations of the forearm, which requires higher control of the forearm. The participation of wrist and finger movements would cause greater disturbance to the arm, which leads to the earlier onset of ECR AMAs for combating the disturbance and improving the task performance. As the onset of several muscles had been used as the predictor of forthcoming movements (3), the task-specific AMA onset latency of ECR might be a useful predictor of the forthcoming wrist and finger movements. The reason why we failed to observe similar changes on muscle FCR may be related to our test paradigms. All tasks were performed in palm-down and palm-inward postures, and the difference in forearm posture may lead to changed AMA onset of both ECR and FCR (9).

However, this task-specific performance in ECR disappeared in stroke subjects even at the non-paretic side (see Figures 3B,D). This phenomenon has been suggested as the obvious deficit of feedforward motor preparation in stroke subjects (55). In previous imaging studies, abnormal hyperexcitability of the bilateral premotor cortex was revealed in stroke survivors, and it had a negative correlation with the recovery of motor function (56–58). As we have learned, the premotor cortex is mainly responsible for motor planning and preparation, and the excessive excitability of these areas would induce reinforced inhibition to the cortical reticulospinal tract (44) and consequently affect the modulation of task-specific AMAs. This was supported by the lower incidence of ECR AMAs and positive SE response, which may reflex the impaired cortical reticulospinal tract after stroke (22, 44). Therefore, rehabilitation interventions for the impaired cortical reticulospinal tract may be helpful for the recovery of AMAs after stroke. For the arm-hand movements, novel treatments could be developed based on the task-specific AMA onset of ECR, such as neurofeedback training for humancomputer interaction and rehabilitation robotics (59).

On the contrary to most previous researches (46), we observed comprehensive earlier AMA onset in ECR, FCR, and bilateral LTs and TAs in stroke subjects. This could not be fully explained by the change of AMAs accompanied by the alignment of the scapula and thorax (60) as mentioned by Pereira et al. (46) in a similar study. One possible explanation is that sufficient time for preparation results in the delayed activation of AMAs in proficient movements. A similar research of Akbaş et al. (54) revealed that the onset of TAs in professional fencers at a well-prepared state was significantly delayed than that of normal people. The time of about 2.5 s, we set, is enough for the subjects to make adequate preparations (61). Another reasonable explanation is that the AMA onset latency of subacute stroke survivors is polarized, with premature or delayed activation onset, and the delayed part has been filtered out by the time window we set and considered as negative AMA response. And a state of full preparation before the motor execution improved the stability of AMA performance in healthy subjects (61) but has limited effects for stroke survivors.

Our research also has some limitations. First of all, we did not use SCM^+^ as the marker to screen valid SE trials for group comparisons. Instead, we included all valid trials that used the startle “Go” cue into the final analysis. In our tests, the positive incidence of SCM^+^ for stroke and normal participants was 16.6 and 25.9%, respectively. The result of healthy subjects in our study has comparable SCM^+^ proportions with the result from a previous study (40) at the same stimuli intensity (114 dB). Such occurrence proportions of SCM^+^ may lead us to underestimate the influence of SE on motor initiation and AMA onset latency or amplitude. Early muscle onset can also be found without premature activation of SCM in trials with startling “Go” cues (62). Simply emphasizing the use of data from positive SCM^+^ trials may lose valuable information from SCM^−^ trials. In the case that the participant is acceptable, a 124-dB cue (39) might be better. The second limitation of our study was that our experiment did not strictly limit the severity of motor impairment of participants. The large difference in FMA score may indicate different dominant neural pathways they rely on (22), which may lead to unstable results. Stroke survivors within different motor function levels may use different motor synergies of flexors and extensors (22) and perform different AMA responses. Stratified analysis should be necessary according to the subject's motor function states in further researches with a higher sample size. At last, our research focused on the initiation of AMAs and did not include classic APA evaluation indicators, such as onset or velocity of displacement of body center of mass on the trunk (3, 5) in this study. SE may affect the movement amplitude and velocity of the trunk and limbs of stroke survivors (30, 31). The integration of a wearable inertial measurement unit with sEMG may better optimize our conclusions. In addition, future research design around upper-limb functional rehabilitation may need to consider more direct and effective evaluation methods, such as grip strength.

## Conclusions

The present research revealed task-specific AMAs of muscle ECR in forward-reaching movements involving precision hand manipulations. This research also indicated the impaired APAs with lower incidence of SE and AMA response, changed AMA onset latency, and smaller AMA amplitude in patients with stroke. And startle can improve the incidence of AMAs in both healthy and stroke populations, but it does not affect the tunes and amplitude of AMA onset. And the deficit of such task-specific AMAs in ECR indicates the impairment of APA programming for arm-hand precision movements on both hands in the stroke population. Finally, these findings may provide support to develop novel methods for APA rehabilitation after stroke.

## Data Availability Statement

The original contributions presented in the study are included in the article/Supplementary Material, further inquiries can be directed to the corresponding authors.

## Ethics Statement

The studies involving human participants were reviewed and approved by Institutional Ethical Committee of Tongji Hospital. The patients/participants provided their written informed consent to participate in this study.

## Author Contributions

XH, JX, and NX: designed the research. NX, CH, YL, MG, ZC, and XW: participated in the subject recruitment, research implementation, and data collection. NX and CH: performed the data analysis. NX: wrote the draft. All authors had full access to the data. All authors have reviewed the research and approved the submitted version.

## Funding

The study was supported by the National Natural Science Foundation of China (Grant Nos. U 1913601 and 91648203).

## Conflict of Interest

The authors declare that the research was conducted in the absence of any commercial or financial relationships that could be construed as a potential conflict of interest.

## Publisher's Note

All claims expressed in this article are solely those of the authors and do not necessarily represent those of their affiliated organizations, or those of the publisher, the editors and the reviewers. Any product that may be evaluated in this article, or claim that may be made by its manufacturer, is not guaranteed or endorsed by the publisher.

## Supplementary Material

Supplementary Material 1 Results of liner model and *post-hoc* comparison of variables. The positive liner model based on data from all subjects and significant main effects of the 3 fixed factors (3 move sides, 2 stimuli conditions, and 3 move tasks) in different variables were provided. The *post-hoc* comparisons with Bonferroni corrections under each fixed factor with significant main effects in its model were provided after each liner model. H, N, and P represent healthy, non-paretic, and paretic sides, respectively. R, B, and C represent the move task reach, reach to grasp a ball, and reach to grasp a cup, respectively. N/A means not applicable.
