# Supplementary material for: Startle Increases the Incidence of Anticipatory Muscle Activations but Does Not Change the Task-Specific Muscle Onset for Patients After Subacute Stroke
Source: Front Neurol. 2022 Jan 13;12:789176. doi: 10.3389/fneur.2021.789176 (PMC8793907; doi:10.3389/fneur.2021.789176)
Supplement: Supplementary Material 1 — Results of liner model and post-hoc comparison of variables. The positive liner model based on data from all subjects and significant main effects of the 3 fixed factors (3 move sides, 2 stimuli conditions, and 3 move tasks) in different variables were provided. The post-hoc comparisons with Bonferroni corrections under each fixed factor with significant main effects in its model were provided after each liner model. H, N, and P represent healthy, non-paretic, and paretic sides, respectively. R, B, and C represent the move task reach, reach to grasp a ball, and reach to grasp a cup, respectively. N/A means not applicable. [file Table_1.docx]

**Supplementary materials 1. Results of liner model and post-hoc comparison of variables.**

| **Variables** | |  | **Liner Models** | **Sides** | **Conditions** | **Move types** | **Post hoc comparison** | | | | | |
| --- | --- | --- | --- | --- | --- | --- | --- | --- | --- | --- | --- | --- |
|  |  |  |  |  |  |  | **Move sides**  (**H**ealthy/**N**onparetic/**P**aretic) Mean (SD) | | ***P values***  （H vs. N,  H vs. P,  N vs. P） | **Movement types**  (**R**each/**B**all/**C**up)  Mean (SD) | | ***P values***  (R vs B,  R vs. C,  B vs. C) |
| **AD Reaction Time** (/ms) | | *F* | 25.07 | 7.87 | 60.68 | N/A | H | 160.47 (68.71) | 1.0 |  | |  |
|  |  | *p* | < 0.001 | < 0.001 | < 0.001 |  | N | 159.80 (79.03) | 0.001 |  | |  |
|  |  | *η^2^_P_* | 0.068 | 0.015 | 0.056 |  | P | 181.51 (93.25) | 0.005 |  | |  |
| **Onset Latencies**  (/ms) | ECR | *F* | 6.66 | 9.85 | N/A | 3.56 | H | -5.85 (44.23) | 0.044 | R | -5.37 (45.91) | 0.483 |
|  |  | *p* | < 0.001 | < 0.001 |  | 0.03 | N | -17.85 (52.80) | < 0.001 | B | -11.76 (46.71) | 0.026 |
|  |  | *η^2^_P_* | 0.040 | 0.030 |  | 0.011 | P | -26.07 (51.12) | 0.536 | C | -17.42 (50.05) | 0.608 |
|  | FCR | *F* | 12.515 | 12.515 | N/A | N/A | H | -4.77 (43.10) | 0.183 |  | |  |
|  |  | *p* | < 0.001 | < 0.001 |  |  | N | -13.65 (53.62) | < 0.001 |  | |  |
|  |  | *η^2^_P_* | 0.036 | 0.036 |  |  | P | -30.15 (58.19) | 0.022 |  | |  |
|  | iLT | *F* | 8.26 | 13.95 | 6.88 | 3.92 | H | -10.84 (52.57) | 1.0 | R | -8.66 (50.03) | 0.273 |
|  |  | *p* | < 0.001 | < 0.001 | 0.009 | 0.020 | N | -9.03 (50.40) | < 0.001 | B | -17.27 (52.42) | 0.028 |
|  |  | *η^2^_P_* | 0.065 | 0.045 | 0.011 | 0.013 | P | -36.37 (48.26) | < 0.001 | C | -22.01 (53.69) | 1.0 |
|  | cLT | *F* | 12.144 | 12.144 | N/A | N/A | H | -8.70 (54.81) | 1.0 |  | |  |
|  |  | *p* | < 0.001 | < 0.001 |  |  | N | -12.50 (51.16) | < 0.001 |  | |  |
|  |  | *η^2^_P_* | 0.051 | 0.051 |  |  | P | -38.00 (51.93) | 0.002 |  | |  |
|  | iTA | *F* | 4.801 | 4.801 | N/A | N/A | H | -22.88 (51.82) | 0.672 |  | |  |
|  |  | *p* | 0.009 | 0.009 |  |  | N | -30.00 (58.05) | 0.007 |  | |  |
|  |  | *η^2^_P_* | 0.017 | 0.017 |  |  | P | -42.50 (55.14) | 0.323 |  | |  |
|  | cTA | *F* | 6.393 | 6.393 | N/A | N/A | H | -20.05 (51.76) | 0.004 |  | |  |
|  |  | *p* | 0.002 | 0.002 |  |  | N | -41.59 (54.11) | 0.087 |  | |  |
|  |  | *η^2^_P_* | 0.026 | 0.026 |  |  | P | -33.43 (55.18) | 0.922 |  | |  |
| **APAs Amplit-udes**  (/times) | ECR | *F* | 8.815 | 8.815 | N/A | N/A | H | 31.57 (29.35) | 0.002 |  | |  |
|  |  | *p* | < 0.001 | < 0.001 |  |  | N | 21.36 (28.14) | 0.006 |  | |  |
|  |  | *η^2^_P_* | 0.026 | 0.026 |  |  | P | 22.51 (24.93) | 1.0 |  | |  |
|  | FCR | *F* | 18.092 | 18.092 | N/A | N/A | H | 23.13 (25.40) | < 0.001 |  | |  |
|  |  | *p* | < 0.001 | < 0.001 |  |  | N | 14.17 (21.71) | < 0.001 |  | |  |
|  |  | *η^2^_P_* | 0.052 | 0.052 |  |  | P | 9.65 (12.82) | 0.403 |  | |  |
|  | iLT | *F* | 8.518 | 12.987 | N/A | 3.807 | H | 26.37 (27.41) | 0.214 | R | 32.50 (31.66) | 0.022 |
|  |  | *p* | < 0.001 | < 0.001 |  | 0.023 | N | 20.95 (20.97) | < 0.001 | B | 24.97 (26.39) | 0.093 |
|  |  | *η^2^_P_* | 0.054 | 0.042 |  | 0.013 | P | 38.47 (35.55) | < 0.001 | C | 26.40 (27.96) | 1.0 |
|  | cLT | *F* | 7.346 | 7.346 | N/A | N/A | H | 39.13 (34.47) | 0.078 |  | |  |
|  |  | *p* | 0.001 | 0.001 |  |  | N | 30.50 (33.56) | 0.001 |  | |  |
|  |  | *η^2^_P_* | 0.027 | 0.027 |  |  | P | 27.60 (27.95) | 1.0 |  | |  |
|  | iLD | *F* | 6.007 | 6.007 | N/A | N/A | H | 41.35 (36.43) | 0.005 |  | |  |
|  |  | *p* | 0.003 | 0.003 |  |  | N | 27.36 (21.53) | 1.0 |  | |  |
|  |  | *η^2^_P_* | 0.023 | 0.023 |  |  | P | 43.62 (35.14) | 0.003 |  | |  |
|  | cLD | *F* | 24.334 | 24.334 | N/A | N/A | H | 44.24 (34.17) | < 0.001 |  | |  |
|  |  | *p* | < 0.001 | < 0.001 |  |  | N | 22.43 (22.94) | < 0.001 |  | |  |
|  |  | *η^2^_P_* | 0.099 | 0.099 |  |  | P | 23.95 (28.68) | 1.0 |  | |  |
